# Hospital Construction

**Published:** 1896-07-11

**Authors:** 


### HOSPITAL CONSTRUCTION.

### MECHANICAL VENTILATION OF HOSPITAL WARDS.

A writer in the *Builder*, who signs himself "F.R.C.S.," urges that the advances which have been made in medical and mechanical science during the past forty years have so altered the conditions of the problem involved in planning a surgical hospital that we may now, by aid of antiseptics and forced ventilation, give up the narrow pavilion plan, and revert to the old "block" plan, as he calls it, namely, wards with four rows of beds between the windows, and a wall down the middle the height of the dado—and all this that we may have some suitable proportion between length, width, and height.

The advantages of such a plan to the architect are, he says, manifest; he can secure "a fine elevation," and the "bugbear of segregation" being dismissed, he may carry his buildings to such a height as the proportions of his plan may require. A little more knowledge would have saved "F.R.C.S." from committing himself to such opinions. It is not air alone that patients gain by the pavilion plan. Light goes for a great deal, and the advantages of the segregation of patients are by no means done away with by the introduction of antiseptics. In fact, the drift of affairs is quite in the opposite direction. Undoubtedly the first triumphs of antiseptic surgery were won in wards crowded with septic cases, and this was important as a proof of the efficacy of the system. But our knowledge of the processes of repair, and of the influence of micro-organisms upon it, has carried us far beyond the stage which "F.R.C.S." seems to have in his mind's eye. Everywhere it is recognised that to get the highest surgical success not only the patient but his whole surroundings should be aseptic. When chemical antiseptics and elaborate dressings were trusted to as a sufficient protection, it may have seemed no great sin to introduce a suppurating wound among a series of operation cases. Now, however, when every attempt is being made to secure asepsis, with as little use of antiseptics, in their old sense, as may be, the segregation of patients is all important, and it is an entire misrepresentation of the facts to say that the principle of segregation and of the pavilion system has now lost its importance. Mechanical ventilation on the plenum system stands on a somewhat different footing. It may be admitted at once that by aid of properly-arranged fans and by conduits of sufficient size, and so arranged as to be capable of being properly and frequently cleaned, a sufficient supply of warmed air can be forced into every ward quite irrespective of external conditions; and it may also be readily admitted, with Lord Kelvin, that the plenum system "approaches as near perfection as human ingenuity can make it." It is to be remembered, however, that this is but partial praise, for perfection has by no means yet been reached, and if human ingenuity can go no farther we must pause before we accept the system with all its present drawbacks.

The problem of plenum ventilation is a question partly of expense and partly of trust in the ceaseless and unfailing perfection of machinery. While the finances of hospitals are such as they too often are, we may doubt whether their managers will provide the money necessary to drive through the conduits day and night the full supply of air required to keep the wards sweet and pure, when by the simple process of opening the windows air equally as pure and fresh can be obtained; nor can we admit that it is prudent, unless forced to it by absolute necessity, to allow the safety of the patients to hinge on the continued and unfailing efficiency of a machine which, like all machines, must at times require stoppages for repairs. When we come to build hospitals on cramped and crowded sites, and to pile ward upon ward in solid blocks of buildings through which no air could permeate unless driven through by force, then we must perforce fall back on mechanical ventilation; but the knowledge that we have this means at our disposal as a last resort should not tempt us to build our hospitals in such a form that they must become unhealthy if these expensive mechanical appliances should fail, or to desert those well-tried methods of construction according to which the air within and the air without are maintained in free and immediate continuity.

